# Chronic Stress-Associated Depressive Disorders: The Impact of HPA Axis Dysregulation and Neuroinflammation on the Hippocampus—A Mini Review

**DOI:** 10.3390/ijms26072940

**Published:** 2025-03-24

**Authors:** Ai Ai Lei, Vanessa Wen Xian Phang, Yu Zhao Lee, Audrey Siew Foong Kow, Chau Ling Tham, Yu-Cheng Ho, Ming Tatt Lee

**Affiliations:** 1 Faculty of Pharmaceutical Sciences, UCSI University, Kuala Lumpur 56000, Malaysia; 2 Faculty of Medicine and Health Sciences, UCSI University, Kuala Lumpur 56000, Malaysia; 3 Department of Biomedical Sciences, Faculty of Medicine and Health Sciences, Universiti Putra Malaysia, Serdang 43400, Malaysia; 4 Natural Medicine and Product Research Laboratory (NaturMeds), Institute of Bioscience, Universiti Putra Malaysia, Serdang 43400, Malaysia; 5 School of Medicine, College of Medicine, I-Shou University, Kaohsiung City 82445, Taiwan; 6 Office of Postgraduate Studies, UCSI University, Kuala Lumpur 56000, Malaysia

**Keywords:** major depressive disorder, HPA axis, chronic stress, neuroinflammation, oxidative stress, human health

## Abstract

Chronic stress significantly contributes to the development of depressive disorders, with the hypothalamic–pituitary–adrenal (HPA) axis playing a central role in mediating stress responses. This review examines the neurobiological alterations in the hippocampus linked to HPA axis dysregulation in chronic stress-associated depressive disorders. The prolonged activation of the HPA axis disrupts cortisol regulation, leading to the decline of both physical and mental health. The chronic stress-induced HPA axis dysfunction interacts with inflammatory pathways and generates oxidative stress, contributing to cellular damage and neuroinflammation that further aggravates depressive symptoms. These processes result in structural and functional alterations in the hippocampus, which is essential for emotional regulation and cognitive function. Comprehending the impact of chronic stress on the HPA axis and associated neurobiological pathways is essential for formulating effective interventions for depressive disorders. This review summarises the existing findings and underscores the necessity for future investigations into intervention strategies to improve physical and psychological wellbeing targeting at HPA axis dysregulation for the betterment of psychological wellbeing and human health.

## 1. Introduction

The World Health Organization (WHO) reported that one in every eight individuals worldwide is diagnosed with a mental disorder [1], including anxiety, depression, bipolar, post-traumatic stress disorder (PTSD), schizophrenia, etc. [2]. In 2020, amid the COVID-19 pandemic, statistics indicated a significant surge in people diagnosed with mental disorders, especially anxiety and depression, with an estimated increment of 26% and 28% respectively, within a single year [1,3]. There might be multiple risk factors, such as biological, psychological, environmental and socioeconomic risk factors, which contribute to the onset of mental disorders, as it is rare for a singular cause to be responsible for a mental illness, and it varies among individuals [4,5]. Depression is regarded as one of the significant concerning issues among all kinds of mental disorders due to its high recurrence and incidence rates in the community [1,4]. Clinical depression, also known as major depressive disorder (MDD), affects cognition, emotions, and behaviour, leading to various medical and psychological issues [6]. Research and development of treatments for MDD have garnered significant attention in recent decades.

Despite ongoing efforts in managing MDD, challenges remain regarding their effectiveness and related adverse effects [7]. Currently available antidepressant drugs predominantly focus on modulating neurotransmitter release and the signalling pathways associated with depression [8]. Several studies highlighted a subset of patients, particularly among children and adolescents, who exhibited improvement without pharmacological intervention; however, a significant number of patients failed to improve despite receiving antidepressant treatment. Thus, it was suggested that the underlying causes of depression extend beyond dysregulated neurotransmitters and signalling molecules [9,10].

This review offers essential insights into the neurobiological mechanisms behind the dysregulation of the hypothalamic–pituitary–adrenal (HPA) axis in chronic stress-induced depressive disorders, with focus on hippocampal dysregulation. Comprehending how chronic stress disrupts HPA axis-mediated cortisol regulation, hence triggering inflammatory and oxidative stress pathways that result in functional brain alterations, is essential for understanding the progression of MDD. As HPA axis dysfunction is critically involved in MDD development, future research towards innovative solutions aimed at mitigating HPA axis dysfunction is warranted for improving mental health outcomes.

## 2. Stress

Stress is a natural reaction to threats or difficulties in our surroundings that facilitates our survival. It activates the body’s stress systems, potentially altering physiological processes [11]. Short-term stressors are referred to as acute stress, whereas prolonged exposure to stressors is indicated as chronic stress. Both acute and chronic stress can affect inflammation and general health [12,13,14]. Research had demonstrated that acute stress rapidly impacts the immune system and inflammatory response [12]. It can alter immunological pathways, boost the synthesis of inflammatory cytokines, and increase the quantity and make-up of circulating leukocytes [14]. On the other hand, chronic stress has been associated with persistent low-grade inflammation and an increased risk of various disorders, including cancer, insulin resistance, and cardiovascular disease, all of which are closely linked with inflammatory processes [13]. Stressor types and their intensity influence both pro- and anti-inflammatory pathways. Acute stressors appear to boost immunity, whereas chronic stressors seem to have the opposite effect. An imbalance between inflammation and anti-inflammation results from intense stressors overstimulating the immune system [15].

Chronic stress is associated with several adverse health effects and disrupted behaviours like exercise, sleep, and balanced nutrition [16,17,18]. Unaddressed chronic stress can lead to significant impairments such as insomnia, elevated blood pressure, anxiety, and muscular aches. It may also be the underlying cause of serious conditions like cardiovascular diseases, obesity, depression, etc. [19]. Chronic stressors can build up and continue, resulting in physiological dysregulation and impaired functionality of various regulatory systems [11].

## 3. Chronic Stress’ Effect on Mental Health

Reports showed that major depressive episodes are strongly associated with stressful life events, while chronic stress is significantly associated with MDD [20,21], which is in line with the literature that found a significant association between psychological stress and depression [22,23]. Additionally, research has demonstrated the deleterious impacts of chronic stress on mental processes and cognitive abilities. The degree, duration, source, and magnitude of stress influence cognition in various manners [24,25]. Moderate stress may enhance cognitive function; however, severe and persistent stress can lead to cognitive disorders, particularly those affecting memory and judgment [26]. Stress affects the brain through hormones and neuropeptides that influence behaviour and cognition, alongside biological effects on various areas of the central nervous system [27].

The pathophysiology of stress-related diseases is notably affected by excessive inflammation, with chronic inflammation as a critical underlying component of many chronic diseases [28]. Stress exposure and the emergence of these diseases may be linked with a shared mechanism of chronic mild inflammation [29]. Activation of the HPA axis and sympathetic system due to stress results in the release of inflammatory mediators and stress hormones [30]. Depressive disorders result from chronic inflammation, where pro-inflammatory cytokine release alters neurotransmission and neuroendocrine function, leading to behavioural, emotional, and cognitive abnormalities [22]. Both acute and chronic stress have an impact on inflammatory activity and emotional attention [31]. Research has demonstrated a positive correlation between negative attentional bias and inflammatory markers, pointing towards a possible neural pathway connecting stress, inflammation, and depression. However, the relationship between inflammation and depression is not consistent across all types of depression, highlighting the need for more specific studies on inflammation’s involvement in different somatic and affective–cognitive conditions [32,33,34].

## 4. Chronic Stress Affects HPA Axis Activity and Cortisol Regulation

Cortisol, a glucocorticoid produced from cholesterol, plays a crucial role in the body’s reaction to stress. The adrenal cortex releases cortisol as the primary glucocorticoid during the “fight or flight” response. The neuroendocrine HPA axis regulates the production and secretion of the hormone glucocorticoids. Hence, HPA axis dysfunction is associated with several pathological mental and physical conditions, including MDD, PTSD, and anxiety, as well as type II diabetes and hypertension [35].

The hyperactivity of the HPA axis may be a primary factor leading to depression, particularly in the context of chronic stress conditions. The body responds to stress by releasing corticotrophin-releasing hormone (CRH) from the hypothalamus. This hormone stimulates the secretion of adrenocorticotropic hormone (ACTH) by the anterior pituitary gland, resulting in cortisol release from the adrenal cortex. Exceeding the threshold level of cortisol triggers the negative feedback mechanism that inhibits the production of CRH and ACTH [36]. Several studies demonstrated that chronic stress disrupts the negative feedback system of glucocorticoids, which leads to abnormal alterations in the higher centres of the HPA axis [37,38]. Hyperactivity of the axis due to changes in glucocorticoid receptors (GRs) lead to abnormally elevated levels of cortisol [39]. Chronic mental or physical stressors may impact health through the altered cortisol levels [40].

Hypothalamic CRH oversecretion or inadequate negative feedback inhibition by endogenous glucocorticoids, resulting from glucocorticoid resistance, is hypothesised to contribute, at least in part, to dysregulation of the HPA axis in depressed individuals [41]. The hypothalamus releases CRH, a 41-amino-acid neuropeptide, in response to stressful events, which further mediates the neuroendocrine, behavioural, and autonomic reactions to stress [42]. Roy et al. (1987) demonstrated that hypercortisolism was correlated with hypersecretion of CRH [43,44]. The findings were supported by a further study conducted by Merali et al. (2004), which compared levels of CRH and the mRNA expression of CRH-binding protein, CRH1, and CRH2 receptors in both depressed suicide victims and a control group. In the frontopolar and dorsomedial prefrontal cortex (PFC), levels of CRH were higher in suicide victims with depression. Suicide brains exhibited reduced mRNA levels for CRH1 receptors compared to CRH2 receptors in the frontopolar cortex, likely due to elevated CRH activity [45]. Pandey et al. (2019) conducted a study on teenage suicide subjects and also revealed similar results, where the post-mortem brain of a suicide subject exhibited significant alterations in the expression of CRH and its receptor. In addition, the study concluded that there are regionally unique changes to CRF and its components, as no alterations of CRF components or protein expression were found in the hippocampus [46].

An animal study using rats with chronic variable stress exposure revealed that impairment of the HPA axis is due to sensitisation rather than desensitisation to rapid glucocorticoid feedback [38]. This is evident from the increased responsiveness of neurons to excitatory inputs and decreased responsiveness to inhibitory inputs in the stress-exposed rats [47]. The findings suggest that the neurons in the hypothalamic paraventricular nucleus exhibit heightened sensitivity to stress-related inputs and signals, resulting in an amplified stress response and elevated ACTH release, which contributes to the observed impairment in glucocorticoid negative feedback. Nonetheless, the researchers found no difference in the inhibition of glucocorticoids by excitatory synaptic inputs between the chronic variable stress-exposed rats and the control rats [38]. This supports the idea that over-secretion of hypothalamic CRH plays a role in the impairment of the HPA axis feedback mechanism.

Chronic stress will induce the excessive release of glucocorticoids, attributed to the prolonged activation of the HPA axis. A higher level of glucocorticoids tends to activate GRs, promoting the expression of a diverse range of genes. This mechanism is believed to mediate the effects of glucocorticoids on inflammatory responses and neuronal functions [48]. In this context, discernible alterations have been identified in the genes encoding the excitatory neurotransmitter glutamate and inhibitory neurotransmitter gamma-aminobutyric acid (GABA) [49]. The brain’s response to stress involves the synthesis of glucocorticoids and glutamate, leading to modifications in synaptic connectivity [50]. These alterations manifest as either dendritic retraction or expansion, accompanied by changes in synapse density, which play a role in modulating the neurogenesis inhibition in the dentate gyrus. The nature of neuronal remodelling is dependent on the specific brain region, particularly evident in the hippocampus, which is responsible for influencing mood and behavioural changes [51].

## 5. Chronic Stress Induces Inflammation

Stimulation of the stress response disrupts the HPA axis, leading to an altered inflammatory system. Several experimental studies demonstrated that this activation enhanced the production of circulating inflammatory cytokines, including interleukin (IL)-6, IL-1β, IL-10, and tumour necrosis factor (TNF)-α [52,53]. Increased levels of pro-inflammatory cytokines in the central nervous system, especially the hippocampus and striatum, are associated with depression [54]. One specific cytokine, IL-1β, activates the kynurenine pathway in human hippocampal progenitor cells and contributes to the reduction of neurogenesis. TNF-α, a pro-inflammatory cytokine, induces excitotoxic damage to surrounding neurons by activating microglia, which in turn promote the release of glutamate. In addition, TNF-α can reduce tight junction protein expression, resulting in the increase of the permeability of the blood–brain barrier (BBB) and leading to larger gaps between the endothelial cells and a consequent loss of BBB integrity. Persistent potentiation in hippocampal neurons, which are responsible for memory storage, will lead to their damage by type I interferons, leading to depressive-like behaviour [55,56].

Previous studies have revealed that acute psychological stress induces the expression of systemic inflammatory cytokines, such as IL-6, IL-10, TNF-α, and IL-1β [57]. C-reactive protein levels were higher in PTSD and anxiety patients than healthy individuals [58]. Studies examining the relationships between depression and the inflammatory markers in peripheral blood revealed a positive correlation between depression and inflammatory markers such as C-reactive protein, IL-1, and IL-6 [59,60]. These cytokines regulate blood pressure and glucose levels, potentially leading to cardiovascular problems and other ailments, including rheumatoid arthritis, psoriasis, and insulin resistance-related diabetes [61,62]. The acute stress reaction is recognised for facilitating the rapid response essential for immediate survival; however, prolonged exposure to stressors can result in chronic stress. Nevertheless, the process through which an initial acute stressor evolves into chronic stress remains inadequately understood [63].

Low-grade inflammation, often termed chronic inflammation, differentiates itself from acute inflammation, such as inflammation from local infection, in many ways [13]. Numerous studies have found that IL-6 activity in chronic inflammation is strongly associated with neuropsychiatric disorders, including MDD. A study demonstrated that Alzheimer’s patients’ caregivers exhibited the highest degree of chronic stress among all groups, as evident by the highest level of IL-6 [64]. These findings were corroborated by Haley et al. (2010), who indicated that caregiver stress significantly impacted the predicted risk of stroke and was associated with early death and the emergence of depressive symptoms [65]. While atherosclerosis is primarily associated with local inflammation, systemic chronic low-grade inflammation plays a role in the development of atherosclerotic plaques at sites of minor arterial injuries and can promote the progression of existing plaques [66]. A study also indicated that myocardial infarction patients that exhibited higher pro-inflammatory cytokine levels were correlated with a higher incidence of depression [67].

Psychosocial stress is a recognised determinant in predicting the progression of depression in humans [68]. Cytosolic protein complexes in myeloid cells, also known as inflammasomes, can respond to both pathogenic microorganisms and non-pathogenic stressors. Psychosocial stress can lead to the activation of inflammasomes, like NLRP3 inflammasome, notably by endogenous damage-associated molecular patterns (DAMPs). The activation of the NLRP3 inflammasome induces the production of pro-inflammatory mediators, leading to a greater severity of depression [69,70]. The increase in glucocorticoids also triggers neuroinflammation. This is evidenced by a shifted immunological response that activates microglia, leading to the continuous release of pro-inflammatory mediators, including prostaglandins and leukotrienes [71]. Microglia activation and the subsequent pro-inflammatory mediator secretion have the potential to generate reactive oxygen species, possibly facilitated by NADPH oxidase and the phagocytosis process, resulting in neuronal tissue damage [72,73]. Post-mortem studies of depression patients revealed activation of microglia and astroglia in multiple brain regions, particularly in the frontal cortex, anterior cingulate cortex, and thalamus [74]. These findings were corroborated by the increased immune responses in the brains of MDD patients, in which the microglia, macrophages, and astrocytes exhibited overexpression of translocator protein (TSPO) [75]. The discovery of cytokines capable of crossing the BBB via humoral, neural, and cellular pathways establishes the notion that a peripheral inflammatory signal can be transmitted to the central nervous system, hence contributing to brain inflammation. Furthermore, TNF released from inflamed hepatic cells induces CCL2 production by microglial cells, leading to monocyte recruitment to the brain and eventual cerebral injury [76,77].

## 6. Oxidative Stress Associated with Chronic Stress

Oxidative stress is attributed to the reactive oxygen species (ROS) production by mitochondria and has been associated with the aetiology of neurological and psychiatric disorders [78]. The brain, being the largest consumer of oxygen and energy and possessing a high concentration of oxidisable lipids, is more vulnerable to damage from excessive ROS than any other organ in the body. In the central nervous system, ROS primarily affect glial cells and neurons, which are more susceptible to damage from free radicals, resulting in neuronal impairment as they are post-mitotic. Consequently, the manifestation of apoptosis leads to the decline of neurons [79]. Persistently high cortisol levels while under chronic stress may promote the production of ROS and aggravate the inflammatory processes. Recent preclinical and clinical studies have shown that increased ROS production and depleted antioxidant defences are integral to the pathophysiology of depression, impacting brain structure [80,81,82].

Chronic stress exposure, by repeated and persistent activation of the HPA axis, promotes oxidative damage [83]. Serum cortisol levels may rise significantly due to oxidative stress [84]. Research suggests that oxidative stress may be a significant biological factor linking mental disorders to HPA axis dysfunction [85].

## 7. Alteration of the Hippocampus Due to Chronic Stress

Numerous studies have highlighted the impact of stress on the hippocampus, which is vulnerable and crucial for regulating autonomic function and the HPA stress response in complex behaviour and cognition. These alterations include dendritic atrophy, synaptic spine loss, suppressed neurogenesis, and volumetric reductions, which collectively impair hippocampal-dependent cognitive processes such as memory formation and emotional regulation [86,87,88,89,90]. The alteration of brain regions is now recognised as a consequence of dendritic remodelling following chronic stress exposure [91]. The activation of glucocorticoid receptors, excitatory amino acid signalling, and corticotropin-releasing hormone (CRH)-mediated pathways collectively disrupt cytoskeletal integrity and synaptic plasticity. Simultaneously, stress-induced epigenetic alterations and the dysregulation of neurotrophic substances such as BDNF exacerbate hippocampus susceptibility. A substantial hippocampal volume reduction in depressed individuals was observed in an extensive imaging study involving 1000 patients with unipolar depression and numerous healthy control participants [92]. Stress and glucocorticoids were identified to be the principal factors contributing to the shrinkage of dendrites and loss of spines in the hippocampus [93].

### 7.1. Dendritic Atrophy and Dendritic Spine Loss in the Hippocampus

Structural and neurochemical alterations, such as the atrophy of the hippocampal neurons and reduced ERK1/2 MAP kinase activity, were also observed in the hippocampi of depressed individuals in a post-mortem study [94]. The morphology of dendrites in the hippocampus, particularly in CA3 pyramidal neurons and dentate gyrus granule neurons, exhibited shrinkage in dendrite length, as well as branch point reduction, relative to the control [95]. The authors revealed that chronic immobilisation stress in rats resulted in significant dendritic atrophy of apical and basal dendrites of CA3 pyramidal neurons, with a more pronounced loss in apical dendrites. Chronic stress induces substantial dendritic retraction in CA3 pyramidal neurons, evidenced by a 20–30% decrease in apical dendritic length and branching complexity [96]. This atrophy is facilitated by glucocorticoid-dependent stimulation of glutamate release, which excessively activates NMDA receptors and disrupts the actin cytoskeleton [97]. Mossy fibre terminals in the CA3 stratum lucidum demonstrate vesicle depletion and mitochondrial accumulation due to persistent restraint stress, indicating increased synaptic activity that leads to dendritic shortening [98]. Parallel reductions in spine density of 15–25% transpire in CA1 and CA3 apical dendrites following stress initiation, a process expedited by CRH–CRFR1 signalling [99]. It was demonstrated that elevated CRH prompted spine retraction by interfering with F-actin polymerisation [99]. Furthermore, intrahippocampal CRFR1 antagonists (e.g., NBI 30775) inhibit spine degeneration and ameliorate memory impairments generated by stress, confirming CRH as a crucial mediator of acute stress effects [100].

### 7.2. Chronic Stress and Adult Hippocampal Neurogenesis

The hippocampus, responsible for memory storage and processing, endures decreases in neuroprotective factors, including brain-derived neurotrophic factor (BDNF) expression and signalling, under chronic stress, which compromises neuronal plasticity [101,102]. The altered hippocampal formation contains both glutamate and mineralocorticoid receptors, indicating that adrenal steroids affect the brain through the hypothalamus. These effects are recognised for impacting regulation of mood and episodic and spatial memory. Blocking NMDA receptors or modulating the ion channels that receive the excitatory signals successfully prevented structural changes in the hippocampus upon stress exposure, akin to the suppression of adrenal corticosterone production [95,103].

Adult hippocampal neurogenesis mostly transpires in the subgranular zone (SGZ) of the dentate gyrus, where radial glia-like neural stem cells (NSCs) produce intermediate progenitor cells (IPCs) that eventually differentiate into adult granule neurons [104]. This process facilitates pattern separation, enhances memory precision, and regulates mood [105]. However, chronic stress was reported to reduce neurogenesis by diminishing BDNF levels by 50% in the dentate gyrus through histone deacetylation at *Bdnf* promoters IV and IX [106,107,108], as BDNF–TrkB signalling is known for its essential role in neural stem cell survival, synaptic function, and maintenance [109]. These signalling changes may be attributed to elevated corticosterone, which binds hippocampal GRs to enhance glutamate release from mossy fibres [110,111]. This activates extrasynaptic NMDA receptors on NSCs, triggering calcium influx and calpain-mediated cleavage of neurogenic transcription factors [112]. Simultaneously, GR signalling downregulates astrocytic glutamate transporters (GLT-1), prolonging excitotoxic microenvironments and leading to neuronal damage in the hippocampus [113,114]. Interestingly, intrahippocampal infusion of BDNF was shown to mitigate stress-induced neurogenic impairments [115] and promote neurogenesis [116].

## 8. Conclusions

**Figure 1:**
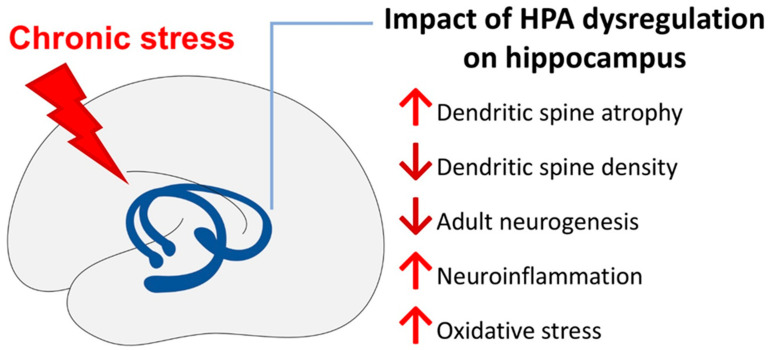
The summary of the impact of HPA axis dysregulation on the hippocampus.

In conclusion, this review highlights the significant involvement of HPA axis dysregulation in chronic stress-induced MDD and its substantial effects on brain function, focusing on the hippocampus. Chronic stress results in HPA axis dysregulation that causes sustained cortisol production. Consequently, this dysfunction leads to neurobiological changes, including neuroinflammation, oxidative stress, and structural alterations in critical brain regions like the hippocampus, as summarised in Figure 1. These changes underpin the emotional, cognitive, and behavioural manifestations associated with MDD. Understanding the mechanism and effects of HPA axis dysfunction provides valuable insights into the pathophysiology of MDD and opens new avenues for targeted therapeutic interventions. Further research is essential to refine treatment strategies aimed at restoring HPA axis balance and improving mental health outcomes in individuals with stress-related disorders.
